# Validation and efficacy of a tele-yoga intervention for improving psychological stress, mental health and sleep difficulties of stressed adults diagnosed with long COVID: a prospective, multi-center, open-label single-arm study

**DOI:** 10.3389/fpsyg.2024.1436691

**Published:** 2024-11-06

**Authors:** Hemant Bhargav, Vijaya Raghavan, Naren P. Rao, Kankan Gulati, Kodikuthiyel Vijayan Binumon, K. N. Anu, Sridhar Ravi, Nishitha Jasti, Bharath Holla, Shivarama Varambally, Padmavati Ramachandran

**Affiliations:** ^1^Department of Integrative Medicine, National Institute of Mental Health and Neurosciences (NIMHANS), Bangalore, India; ^2^Department of Psychiatry, Schizophrenia Research Foundation, Chennai, India; ^3^Department of Psychiatry, National Institute of Mental Health and Neurosciences (NIMHANS), Bangalore, India

**Keywords:** yoga, COVID-19, stress, anxiety, depression, insomnia

## Abstract

The objective of this study was to validate and test the efficacy of a 16-week tele-yoga intervention for perceived stress, anxiety, depression, and insomnia in individuals who had had COVID-19 infection in the previous year, and had reported moderately high levels of psychological stress (PSS ≥14). 25 and 50-min versions of the program were developed. They were then validated using Lawshe’s content validity ratio after obtaining feedback from 20 yoga therapy experts. The safety and efficacy of the two programs were subsequently tested in a prospective, multicenter, open-label single-arm study. Eighty-six adults (18 male, 68 female) were recruited from two tertiary mental healthcare institutions, 48 in NIMHANS, Bengaluru; and 38 in SCARF, Chennai. Participants were assessed at weeks zero, 4, and 16 using validated tools. Data were analyzed using a Mixed Model, Intention to Treat approach. After week 16, 31 subjects remained in the trial and continued to practice yoga without any side effects. Subjects who adhered in the trial had significantly higher levels of baseline anxiety and depression as compared to subjects who dropped out. Results at week 4 included significant reductions in levels of perceived stress, anxiety, and insomnia; improvements were maintained at week 16. Correlations between number of yoga sessions and post-intervention PSS scores were negative (r = −0.49), and significant (*p*<  0.05). Both tele-yoga programs proved safe, useful tools to counteract perceived stress, anxiety and insomnia. Future trials should explore the utility of tele-yoga as a tool to enhance well-being and manage stress.

## 1 Introduction

Many countries continue to report infections by SARS-CoV-2, the virus that caused Coronavirus Disease 2019 (COVID-19). A further condition may continue to affect people after recovering from COVID-19, namely Long-COVID syndrome (LC). This term was introduced to describe a set of disorders that persist or occur over 4 weeks after the elimination of SARS-CoV-2 from the body (Akbarialiabad et al., 2021). A systematic review of 33 studies reported that, even after recovery from COVID-19, patients reported psychiatric symptoms affecting their long-term quality of life (Marchi et al., 2023). The most common manifestations were sleep disturbances, followed by depression, post-traumatic stress, anxiety, and cognitive impairment. Sustained long-term psychological illnesses observed post-recovery from acute corona virus infection, include: psychological distress; post-traumatic stress disorder; and depression, (Park et al., 2020).

Various interventions have been tested to mitigate the resulting pain of isolation and stigma; the internet has proved a boon for such purposes. Aside from medications, psychotherapy, tele-counselling services and cognitive therapies, have played important roles in decreasing people’s plight. But, more intense and efficient interventions, that can enhance the ‘sense of connectedness’ are necessary to recover mental wellbeing (Bhargav et al., 2023). Yoga, including yoga meditation, offers interventions with the power to improve the lives of vulnerable people in situations of this kind. All yoga therapy practices aim to improve overall health by aligning a person’s bio-rhythm with that of nature (Svātmārāma, 1975). In addition to behavioral components, yoga therapy also uses physical postures (asanas), life-breath expansion (pranayama), and meditative techniques (dharana, dhyana and samadhi; Varambally et al., 2021).

Yoga has proven better than physical exercise in view of its mindfulness principles and has yielded better outcomes in clinical trials (Ramajayam et al., 2016). Yoga has reduced recidivism in correctional settings of both adolescents and adults (Kerekes et al., 2017); by virtue of its ease of practice, and the possibility of delivering it to groups, it is now emerging as a universally acceptable means to manage stress.

The mechanisms behind yoga’s ability to provide its observed psychological impact has also been researched, viz.: effects on the posterior hypothalamus, salivary cortisol levels, and autonomic nervous system modulation. Its psychological mediators are positive affect, self-compassion, mindfulness and sense of connectedness (Riley and Park, 2015).

Tele-yoga services ease logistic difficulties of treating patients in phases of isolation and other disruptions to normal life. Such services use remote contact modality that increases connectedness and reduce perceived social isolation. Tele-yoga services for chronic illnesses such as COPD, chronic pain, and heart disease have proven efficacious in normal populations (Selman et al., 2015; Donesky et al., 2017; Mathersul et al., 2018). Learning yoga asanas and yoga pranayama is easy; the techniques involved are usually simple enough for beginners. Asanas are also capable of building physical endurance. Such simplicity makes yoga an ideal candidate for e-learning and practice. Yogasanas can fill the void created by lack of physical exercise and the results of isolation policies. No equipment is required for yoga practice; no barrier for the common man. The spiritual aspect of Yoga interventions will also have a positive impact on mental health adding to collective efforts towards empowerment of mankind. The Ministry of AYUSH recently released tele-yoga guidelines setting safety norms.^1^

At the NIMHANS Integrated Centre for Yoga, the authors conducted online yoga for people confined to home under lockdown (Jasti et al., 2020). However, that protocol was not scientifically validated, nor had it been tested for its potential utility. A literature search revealed the need for a scientifically validated, scalable, tele-yoga intervention; one that is both safe and able to reduce stress and enhance overall mental health in adults; especially those with a history of trauma who report continuing significant levels of stress. The current study aims to validate a tele-yoga module specifically designed for stress reduction and to evaluate the effects of a 16-week tele-yoga stress management program through an open label multicentric trial. The primary focus is on perceived stress, with secondary outcomes including anxiety, depression, insomnia, general health, and trauma-related distress. This research targets adults who have experienced COVID-19 within the past year and report moderate psychological stress levels. By investigating the efficacy of this tele-yoga intervention, the study seeks to provide a scientifically backed resource for enhancing mental well-being in this population.

## 2 Materials and methods

### 2.1 Development

For the first phase of the study, development of the yoga-based intervention, literature searches of both traditional and contemporary yoga texts were conducted: traditional texts: (1) *Hatha Yoga Pradipika*, (2) *Hatha Rathnavali*, (3) *Yoga Vashishtha*, and (4) *Patanjali Yoga Sutras*; contemporary texts included Principles and Practice of Yoga in Health Care, Yoga as Medicine, Science and Art of Yoga in Mental and Neurological Healthcare, Yoga for Promotion of Positive Health, and Yoga for Anxiety and Depression.

Traditional Yoga considers stress and its consequences in terms of imbalances arising from excess *Vata* and *Pitta Doshas*, physically, and excess *Rajas Guna*, mentally. We used key words “*Vata*,” “*Pitta*,” ‘*Rajas*,” “*Raga*” (attachment/craving), “*Bhaya*” (fear), “*Vikshepa*” (agitation), “*Anidra*” (lack of sleep), “*Klesha*” (mental afflictions), and “*Dukkha*” (pain) to identify relevant sections in the literature. Next, searches of the modern literature were conducted using: Google Scholar; PubMed; and PsychInfo. Keywords “*Pranayama*,” “*Yoga*,” “*Hatha Yoga*,” “Relaxation,” “Meditation,” “Stress,” “Burn Out,” “Sleep,” and “Fatigue” were employed. Complex and advanced postures, practices with no clear description, or contra-indicated in painful conditions were excluded. The yoga modules were thus designed to include *Asana* preparatory practices, *Yogäsanas* (physical postures), *Pranayäma* (Breathing practices), relaxation techniques and *Dhyana* (meditative practices).

### 2.2 Content validation

The resulting preliminary yoga modules were given to experts with a doctoral degree (MD or PhD) in Yoga for evaluation. The experts had to have either 5 years clinical experience managing patients with substance use disorders by Yoga therapy, or a Master’s degree in Yoga with 7 years clinical experience. They were contacted by phone, and briefed about the study before their consent to participate was sought. The yoga module was sent by email for evaluation to 20 who consented. Of these 10 responded. They were provided with details of stress-related symptoms and asked questions pertaining to the usefulness and contra-indications of the selected practices in managing symptoms of stress. The focus was on selecting practices to reduce over-activity of the SNS, improve sleep quality, and overall well-being.

Of these, 10 responded with their feedback and scores for various practices in the module for calculating the content validity ratio (CVR). For assessing validity of practices, content validity ratio (CVR) was determined using Lawshe’s formula. The CVR for the total number of items in the module was computed based on the experts’ validation. According to Lawshe’s formula, if more than half of the experts indicate that an item is essential, then that item has the minimum content validity. The CVR cut off was thus ≥0.6, which was considered satisfactory for a panel of 10 experts based on Lawshe’s cut-off table.

Practices with CVR scores ≥0.6 were included in the module. Further, expert opinions and suggestions were asked on the duration, appropriateness and sequence of the practices. Experts were also asked to evaluate practices for their utility (usefulness or potential harm). Opinion was asked from experts for two versions of the yoga for stress management program: a longer one of 50 min duration, and a shorter one lasting 25 min. Table 1 below lists all practices in the final validated modules. Including CVRs for both.

**Table 1:** List of yoga practices for stress management obtained from literature search (development phase) with content validity ratio scores and comments from the experts.

S.N.	Yoga Practices	Ne	N/2	CVR	Remarks/Expert suggestions	Recommendation for the long morning practice module by more than 50% of experts	Recommendation for a brief sos practice module by more than 50% of experts
A. Physical Postures and Practices							
1	Tadasana stretch and side bending	10	5	1	Retained	Yes	Yes
2	Hands in and out breathing	10	5	1	Retained	Yes	No
3	Forward and Backward Bending *(Ardha-chakrasana, Padahastasana)*	10	5	1	Retained with addition of *Humming during forward bend*	Yes	Yes
4	*Vrikshasana* (Tree pose)	4	5	−0.2	Deleted due to probable difficulty in balancing and not recommended on tele-mode	No	No
5	*Surya Namaskara* (Sun Salutations; 10 steps each round): 4 cycles slow, 4 cycles fast	4	5	−0.2	Deleted due to difficulty in implementing the same through tele-mode and potential risk of fall and contraindications in back pain and heart disease.	No	No
6	Instant relaxation technique (quick tightening and relaxation of body parts with breath synchrony)	4	5	−0.2	Deleted for long practice module but retained for brief SoS practice module as advised by experts	No	Yes
B. Breathing Practices (Pranayama)							
7	*Vibhagiya Pranayama* (Sectional Breathing: Breath in: hold: breath out = 4:16:8) in *chin mudra, chinamaya mudra* and *adi mudra*	8	5	0.6	Retained	Yes	No
8	*Ujjayi Breathing* (Victorious breath)	2	5	−0.6	Deleted, difficulty in teaching online	No	No
9	*Kapalbhati* (Skull shining breath)	9	5	0.8	Retained with reduced strokes (30 strokes per cycle for 1 cycle followed by *bahya kumbhaka*)	Yes	No
10	Bhahya Kumbhaka (external retention of the breath)	9	5	0.8	Retained	Yes	Yes
11	*Bhastrika* (Bellows breathing; 3 cycles 30 each)	8	5	0.6	Retained with reduced intensity (30 per stroke for 1 cycle)	Yes	No
12	*Nadi shuddhi* (Alternate Nostril Breathing)	10	5	1	Retained	Yes	Yes
13	*Sitali Pranayama* (Cooling breath)	2	5	−0.6	Deleted, not recommended, may aggravate *vata*	No	No
14	*Chandra Anuloma Viloma* (Left nostril breathing) *Pranayama*	4	5	−0.2	Deleted, not recommended for low mood symptoms	No	No
15	*Bhramari* (Humming breath)	8	5	0.8	Retained, As an alternative to Om chants	Yes (as part of *Nadasnusandhana*)	Yes
C. Meditative/Relaxation Practices (Dhyana)							
16	Deep abdominal breathing in shavasana (I:E = 1:2)	8	5	0.6	Retained	Yes	No
17	*Nadasandhana (A, U, M chants)* and OM chanting	10	5	1	Retained; recommended to chant only humming sound ‘mmm’ if subject is unwilling to chant Om, recommended to chant Om intermittently after each practice	Yes	Yes
18	Guided yogic relaxation (modified *yoga nidra*) in Shavasansa (corpse pose), with positive affirmation—slow part-by-part relaxation of body parts with imagery from toes to head, with chanting, positive affirmation: “I am not the body, I am not the mind.” This was followed by gradual expansion of the non-judgmental awareness.	10	5	1	Retained	Yes	Yes (brief version)
19	Discussions on Yoga Philosophy (once a week for 10 min): *Pancha kosha model*, understanding stress through yoga perspective and coping of stress through yoga practice and philosophy, happy analysis (*ananda mimamsa*) and inquiry into the nature of the “self” (*aatma parikasha*), choosing the most suited stream of yoga (*jnana, karma, kriya, or bhakti*) and channelizing energy in that direction.	10	5	1	Retained	Yes	No

### 2.3 Final validated yoga intervention (tele-yoga program)

Based on the expert feedback, two versions of the TYP were developed: a longer, 50 min one for daily morning practice on an empty stomach,^2^ and one of 25 min for practice as and when required.^3^ The longer version included; 15 min of warm-up practices, with postures performed with mindfulness; 20 min of pranayama (incorporating both fast and slow breathing practices); ending with 15 min of guided yogic relaxation (modified yoga nidra).^4^ The longer version of the tele-yoga program also included a weekly 10-min discussion on yoga philosophy described in ancient texts of yoga emphasizing its helpful understanding of health and disease, its principles which help resolve emotional conflicts.

### 2.4 Feasibility, safety, and potential efficacy testing

#### 2.4.1 Study sites

The research project was conducted at two centres, Schizophrenia Research Foundation (SCARF) in Chennai, and at NIMHANS in Bengaluru, at its Department of Integrative Medicine, Integrated Centre for Yoga.

#### 2.4.2 Sample size

Required sample size was calculated using G*power analysis; the outcome indicator was perceived stress level score. Our previous pilot study (Jasti et al., 2020) observed an effect size of 0.89 for tele-yoga in reducing stress levels; so, setting α at 0.05 and β power at 0.90, yielded a sample size of 43. Experience in the pilot study suggested an attrition rate around 50% after 16 weeks, so our sample size was set at 86 subjects.

#### 2.4.3 Study design

A prospective, multi-centric, open-label, single arm exploratory study.

#### 2.4.4 Study participants

Specific measures were taken to recruit COVID-19 affected subjects. The investigators worked with the state government. A list of people affected by COVID-19 infection was accessed to recruit subjects for participation in the study, who were subsequently contacted for a comprehensive telephone interview. Information about the study was given, and eligibility for the trial assessed.

#### 2.4.5 Selection criteria

For inclusion, individuals, belonging to all genders, with a COVID-19 positive report from an NABL approved laboratory, who also reported at least a moderate level of stress (a score ≥ 14 on PSS) were considered. Only those aged 18 to 60 years were recruited. Exclusion criteria were: (1) Anyone practicing yoga or meditation in the past 1 year; (2) Physical or psychological limitations preventing light physical activity (yoga postures). Those reporting symptoms of suicidal ideation, self-harm, or psychosis as assessed by a psychiatrist at a clinical interview were also excluded; as were those with serious or complicated co-morbidities like uncontrolled hypertension or diabetes, heart disease, chronic lung/liver/kidney disease, or cerebrovascular disease.

#### 2.4.6 Outcome measures

Primary effectiveness outcome: Fixed assessment time points were: weeks 0, 4 and 16, for all subjects for pathology assessments. Yoga performance was also assessed at 1 week. Pathology assessments were for the following variables: perceived stress, general psychological health, anxiety, depression, sleep quality and distress due to traumatic events.

The Perceived Stress Scale (PSS; Cohen et al., 1983) is a 10-item scale measuring the degree to which one’s life situations are seen as stressful. Items are rated on a 5-point Likert scale ranging from 0 (never) to 4 (very often). The PSS has shown good internal consistency: α being 0.80 to.86 (Nordin and Nordin, 2013).

The General Health Questionnaire (GHQ; Goldberg, 1988) comprises 12 items, each assessing the severity of a psychological problem over the past few weeks using a 4-point scale (0–3). The GHQ also has high internal consistency with similar α values, 0.82–0.86 (Goldberg, 1988).

The Hospital Anxiety and Depression Scale (HADS; Zigmond and Snaith, 1983) is a 14-item scale used to assess levels of anxiety (7 items) and depression (7 items), scored from 0 to 3. Its psychometric properties are well-established; mean α coefficients for anxiety and depression subscales being 0.83 and 0.82, respectively (Bjelland et al., 2002).

The Insomnia Severity Index (ISI; Morin, 1993) is a 7-item screening measure of insomnia. Items assess difficulty of sleep onset and severity of difficulty in staying asleep, satisfaction with current sleep pattern, interference with daily functioning, appearance of impairment attributable to sleep problems, and degree of concern caused by insomnia. Items are rated on a 5-point Likert scale from 0 (not at all) to 4 (extremely). The ISI has good internal consistency Cronbach’s α, 0.76 (Bastien et al., 2001).

Impact of Event Scale-Revised (IES-R; Christianson and Marren, 2012) is a 22-item self-report measure assessing subjective distress caused by traumatic events. Items are rated on a 5-point scale ranging from 0 (not at all) to 4 (extremely). The IES-R yields a total score ranging from 0 to 88, while subscales concern Intrusion, Avoidance, and Hyperarousal. The IES-R shows good internal consistency (Creamer et al., 2003).

Intervention adherence: Tele-monitoring was done and adherence to the yoga intervention was assessed and documented (Jasti et al., 2020).

Safety: Safety was assessed by documenting numbers of side effects of the yoga practices, and the frequency of each side effect reported by the subjects.

#### 2.4.7 Ethics approval and considerations

Institutional Ethics Committee (IEC) approval was obtained at both research sites (NIMHANS and SCARF; NIMHANS/HECAIM/ 2^nd^ Meeting/2020–21 dated March 24^th^ 2021) before the start of the study. In accordance with ethical regulations for research involving human subjects, informed consent was obtained from all participants by telephone, and e-signatures on an electronic consent form (ex: email/web-based communication). Participants were informed the study is voluntary and of their right to drop out for any reason without any adverse consequence. Each participant was assigned a personal research number to preserve confidentiality on study-related documentation. The study was registered under the Clinical Trial Registry of India as Trial # CTRI/2021/09/036737.

#### 2.4.8 Intervention

The intervention consisted of the 50-min validated TYP taught by trained yoga therapists at each site through a video conferencing facility, to groups of 20 participants or less at a time. Ten supervised tele-yoga sessions were followed by home practice, using a recorded video of the yoga module. On completing the 10 supervised tele-yoga sessions, subjects were asked to practice on their own, at least 5 days a week, using the said video. They were also encouraged to practice the shorter, 25-min version of the module as and when required (based on subjective feeling of psychological stress), in addition to their practice of the longer version early in the morning between 6:50 am to 7:40 am from Monday to Friday. YouTube links of the respective yoga modules was also shared with them.^5^

Each Yoga participant was followed up weekly, by a telephone call to record the number of times he or she had practiced at home that week. They were also asked to keep a diary recording the number of yoga sessions they did each day. A monthly online supervised session observed their performance to correct their practices, as needed.

#### 2.4.9 Data management

Data collected were kept confidential in participant case report forms (CRFs), and later transferred into electronic form. The principal investigator was responsible for maintenance of safely kept records and back-up of data.

#### 2.4.10 Statistical analysis

R programming language was used to analyze data. Since this was a longitudinal clinical trial with missing values, we used a Mixed Model Approach to an Intention to Treat analysis.

## 3 Results

### 3.1 Baseline demography

The 86 subjects had a mean age, 37.38 ± 13.19 years. Being an inclusion criterion, all subjects reported moderate to high PSS stress scores at baseline, mean ± SD was 22.65 ± 5.31. Other variables: 60.4% reported clinical anxiety, HADS anxiety score ≥ 8; 45.34% reported clinical depression, HADS depression score ≥ 8; and 55.8% reported at least sub-threshold insomnia, ISI score ≥ 8. The Impact of Event Scale found that 38.37% of the subjects had significant distress due to trauma, IES-R > 24. 46.5, 26.7 and 22% of subjects, respectively, reported anxiety, depression, and insomnia of moderate to severe intensity. Comparison of baseline demographics between subjects who dropped out and subjects who remained in the trial found that their ages and genders were comparable. However, those who remained in the trial had significantly more distress, general health problems, and anxiety and depression, compared to the dropouts (Table 2). This suggests that subjects starting with less acute psychological and physical health issues felt less need for continued yoga practice, while those with more severe symptoms appreciated it more.

**Table 2:** Comparison of baseline demographic data between subjects remaining in the trial and dropouts by 16 weeks.

Variable	Subjects remaining in trial	Subjects dropping out at 16 weeks	*p* value^a^
*N*	31 (24 females, 77.4%)	55 (44 females, 80%)	
Age (years)	37.34 ± 13.06	37.54 ± 13.65	0.81
Education (years)	17.34 ± 7.06	16.54 ± 8.65	0.21
Employment status	Yes (*N* = 20) Work from home (*N* = 18) No (11)	Yes (*N* = 41) Work from home (*N* = 33) No (10)	
PSS scores	23.96 ± 4.97	21.86 ± 5.40	0.09
GHQ	19.45 ± 7.43	12.12 ± 10.92	0.01**
HADS Anxiety	9.89 ± 5.37	6.32 ± 6.29	0.04*
HADS Depression	7.87 ± 4.78	5.19 ± 5.89	0.04*
ISI	10.89 ± 6.82	7.06 ± 8.29	0.03*
IES-R	22.10 ± 19.25	18.61 ± 20.27	0.01**
Total number of yoga sessions practiced - longer version (during the study period)	51.0 ± 9.5	20.0 ± 16.5	0.01*
Total number of yoga sessions practiced - shorter version (during the study period)	14 ± 5.6	6 ± 2.7	0.01*

a Independent *t* test. *p < 0.05; **p < 0.01.

### 3.2 Post-intervention results

**Figure 1:**
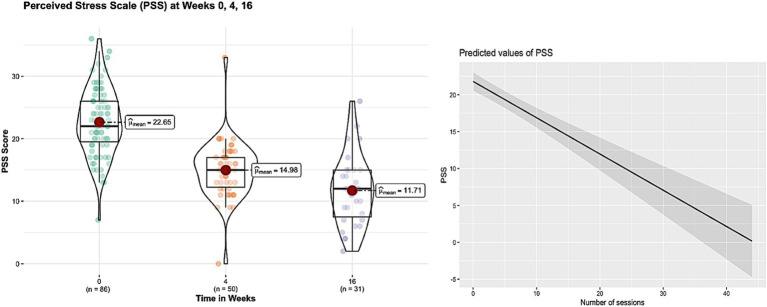
Figure showing the changes in perceived stress scale scores before and after 16 weeks of yoga intervention and significant negative correlation (r = −0.49, *p* < 0.01) of number of yoga sessions with perceived stress scores.

**Figure 2:**
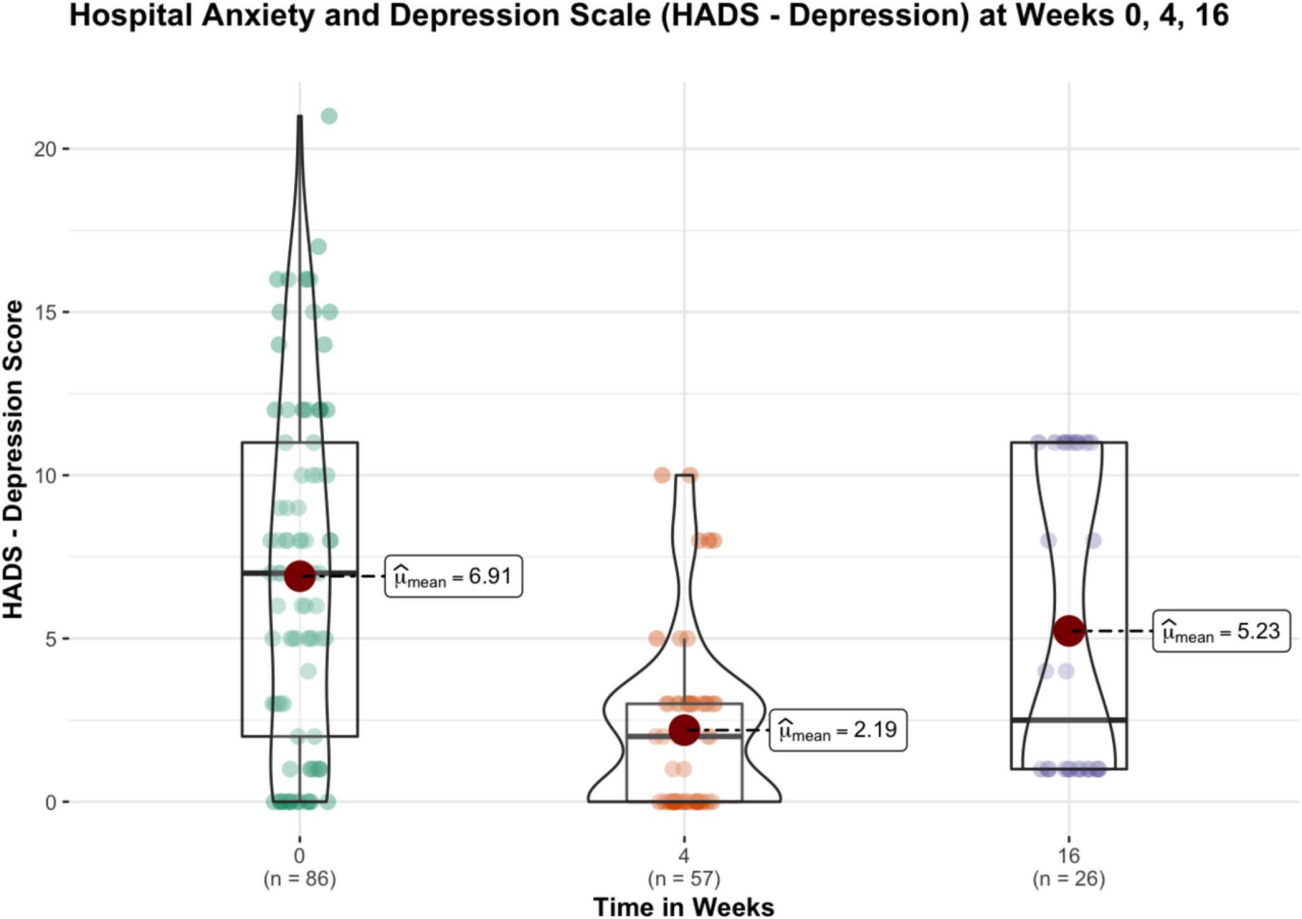
Figure showing the changes in depression scores (HADS—depression) before and after 16 weeks of yoga intervention.

**Figure 3:**
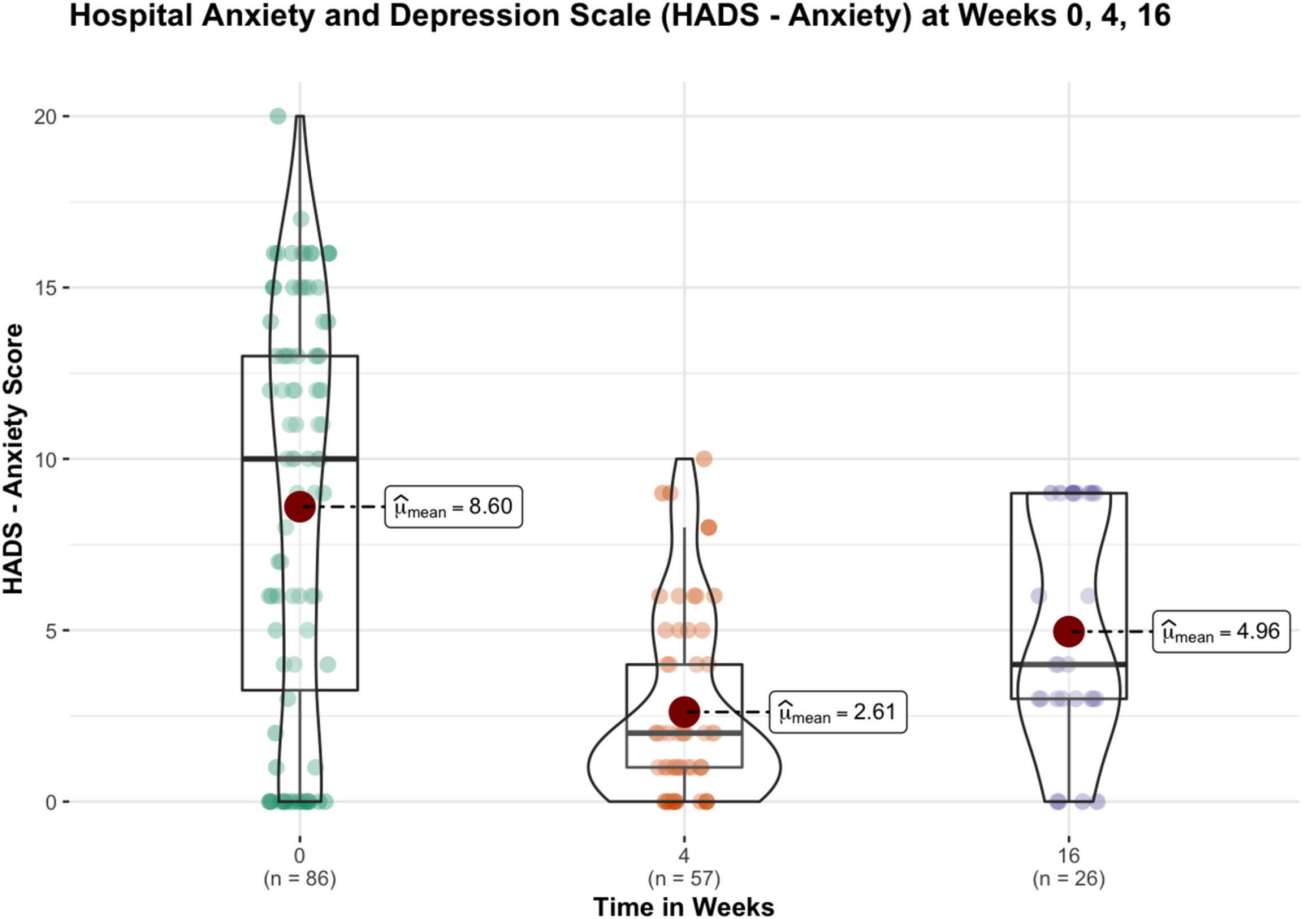
Figure showing the changes in anxiety scores (HADS—anxiety) before and after 16 weeks of yoga intervention.

**Figure 4:**
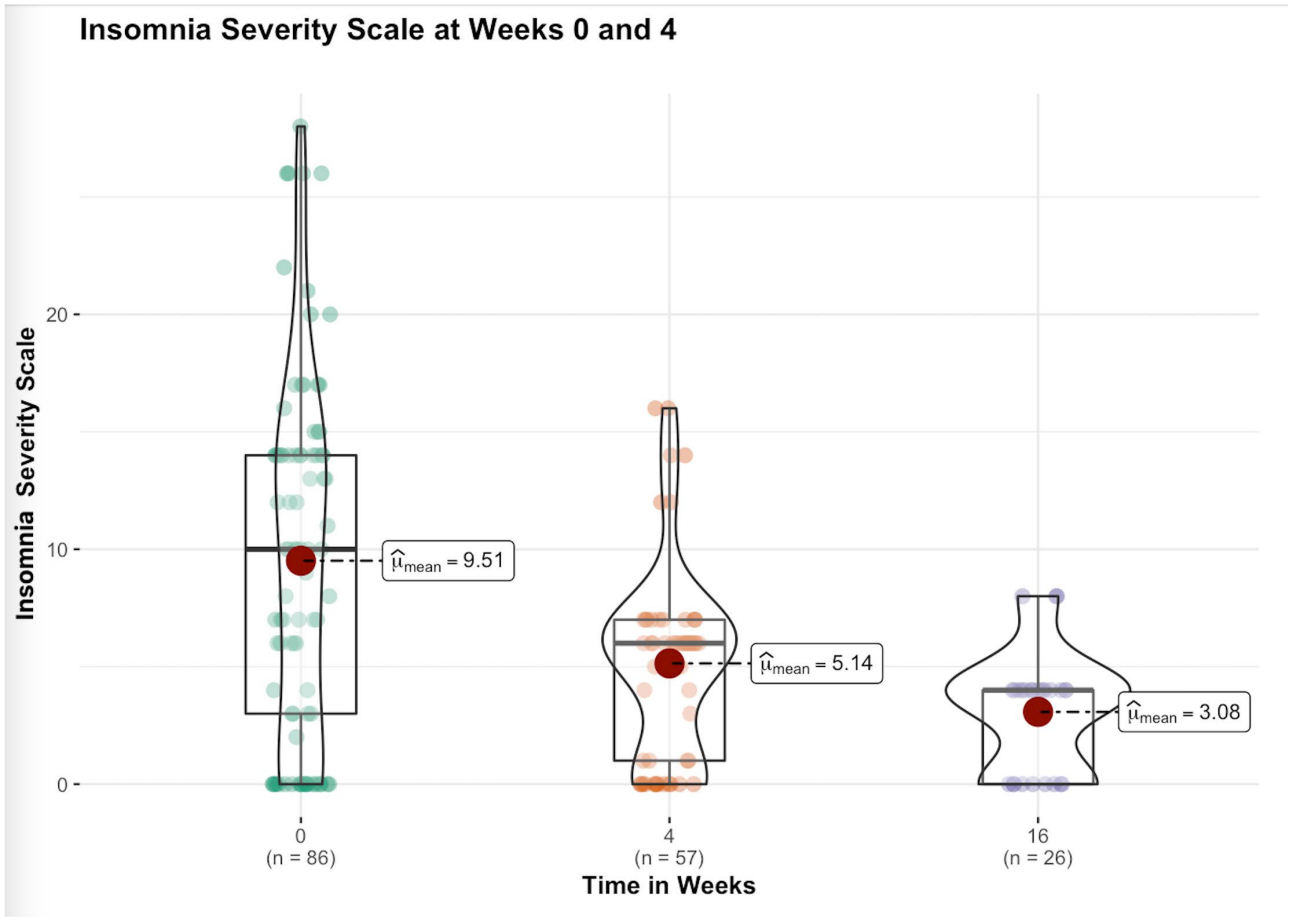
Figure showing the changes in insomnia severity scale scores before and after 16 weeks of yoga intervention.

After week 16^th^, 31 subjects remained in the trial and continued their yoga practice. Significant reductions in perceived stress levels were observed at week 4 and 16 compared to baseline. General health, anxiety and insomnia improved significantly at week 4, and the improvements sustained at week 16. Depression and Distress Due to Trauma scores significantly reduced at week 4 but failed to show significant reductions at week 16. Table 3 provides details of these results, while Figures 1–4 put them into graphical form. Significant (*p* < 0.05) negative correlations were observed between number of yoga sessions that subjects participated in, and post-intervention scores in: PSS (r = −0.49), GHP (r = −0.45), HADS anxiety (r = −0.16), and ISI (r = −0.23), respectively.

**Table 3:** Results of the teleyoga program on mental health variables at weeks 4 and 16.

Variables	Week 4 (T2)				Week 16 (T3)			
	Estimated mean and CI	Standard error	*t* value	*p*^a^ value	Estimated mean and CI	Standard error	*t* value	*p*^a^ value
PSS	−7.67 −9.49, −5.85	0.92	−8.33	< 0.001	−10.94 −13.08, −8.80	1.08	−10.10	< 0.001
GHQ	−12.07 −14.62, −9.52	1.29	−9.34	< 0.001	−12.74 (−16.04, −9.43)	1.67	−7.61	< 0.001
HADS depression	−4.68 −6.15, −3.21	0.75	−6.27	< 0.001	−1.60 −3.55, 0.35	0.99	−1.62	0.10
HADS anxiety	−5.98 −7.56, −4.40	0.80	−7.47	< 0.001	−3.61 −5.68, −1.53	1.05	−3.43	< 0.001
Insomnia severity scale	−4.37 (−6.40, −2.34)	1.03	−4.26	< 0.001	−6.43 (−9.09, −3.78)	1.35	−4.78	< 0.001
Impact of event scale	−10.50 −16.15, −4.86	2.86	−3.67	< 0.001	1.75 −5.73, 9.22	3.79	0.46	0.645

PSS, perceived stress scale; GHQ, general health questionnaire; HADS depression, hospital anxiety depression scale—depression scores; HADS Anxiety, hospital anxiety depression scale—anxiety scores. *Pearson’s correlation coefficient.

a Mixed model approach with intention to treat.

Qualitative feedback documentation from subjects regarding safety of the module revealed that only 3 subjects (3.70%) reported untoward effects (giddiness due to *bhastrika* pranayama by 2; fall during *ardhachakrasana* by 1) due to the tele-yoga practice. Subjective feedback assessment regarding factors affecting adherence to tele-yoga practice showed that “busy schedule (lack of time)” was the main reason for the subjects’ inability to adhere to the yoga program (reported by 67% of subjects who dropped out) other subjects did not provide any answer or they were not reachable at all. 34% of subjects who dropped out preferred to practice yoga using YouTube video at their convenient time rather than the fixed live sessions. Comparison of change in perceived stress scores from baseline to 4 weeks (post minus pre) among subjects who dropped out after week 4 and subjects who continued to be in the trial between week 4 to week 16 revealed that subjects who dropped out after week 4 had significantly greater reduction in stress levels as compared to subjects who continued in the trial (mean ± SD: −9.85 ± 6.56 vs −6.35 ± 4.2; *p* < 0.05, Mann Whitney U test).

## 4 Discussion

We observed that there was a significant reduction in stress levels, anxiety and insomnia and significant improvement in general health by the end of 16th week. Though there was a significant reduction in depression and distress due to trauma at 4 weeks the effects did not sustain till 16th week. We also observed moderately strong correlation between reduction in stress scores and improvement in general health and number of yoga sessions, respectively. Similarly significant but mild correlations were observed between reduction in anxiety scores and insomnia severity with the number of yoga sessions, respectively.

Ruchi et al. (2023) recently reviewed the effects of tele-yoga during the COVID-19 pandemic, synthesizing data from seven randomized controlled trials. Their findings indicated that tele-yoga is both feasible and beneficial for various populations, improving symptoms such as dyspnea and psychological burdens, including stress, anxiety, and depression. Notably, the majority of the studies included in their review focused on specific health conditions, such as cancer, heart failure, and chronic obstructive pulmonary disease. One trial also addressed PTSD in women, while another examined the well-being of individuals working from home during the pandemic (Wadhen and Cartwright, 2021). Wadhen and Cartwright’s pilot randomized controlled trial highlighted the feasibility of a 6-week online yoga intervention for those working from home, finding significant improvements in perceived stress, mental well-being, and depression. However, they did not observe significant changes in stress and anxiety as measured by the DASS-21. In contrast, our study demonstrated significant reductions in both perceived stress and anxiety at the 4-week and 16-week marks. Interestingly, we did not find a sustained reduction in depression by week 16. One possible explanation for this discrepancy lies in our study’s inclusion criteria; we focused specifically on participants reporting moderate to high levels of psychological stress at baseline. Among our subjects, approximately 60% experienced anxiety, with 45% classified as having moderate to severe anxiety. In contrast, only 22% experienced moderate to severe depression. This higher baseline prevalence of anxiety likely provided a greater opportunity for reduction through yoga, while depression symptoms may have shown limited change due to a ceiling effect in this population. Shukla et al. investigated the impact of various yogic breathing practices during the lockdown, finding that specific techniques, such as anuloma viloma, significantly reduced perceived exertion even after just 1 week of practice (Shukla et al., 2020). Our study extends this research by focusing on a population with moderate to severe stress and implementing a longer intervention period of 16 weeks.

Wadhen and Cartwright focused specifically on Hatha yoga practices delivered via tele-mode, while Shukla et al. utilized breathing interventions exclusively. In contrast, our trial implemented a more generic tele-yoga program that combined simple postures, breathing practices, and relaxation techniques, with the added flexibility of being performed while seated. This approach was previously pilot tested during the pandemic (Jasti et al., 2020), ensuring that our program was both accessible and practical for participants. Additionally, we dedicated 10 min each week to discussions on yoga philosophy and the yogic understanding of stress. This educational component is crucial, as it helps participants recognize that while they may not have control over external situations, they can gain control over their mental responses through consistent yoga practice. For instance, one key principle we emphasized was that by training the mind, individuals can shift their perspective on stressors, thereby enhancing their ability to cope. This holistic approach not only provided physical benefits but also fostered a deeper understanding of the psychological tools available through yoga. By integrating philosophy with practice, participants were better equipped to apply these concepts to their daily lives, potentially leading to more profound and lasting changes in their stress management. Our findings suggest that this multifaceted tele-yoga intervention may offer a comprehensive strategy for promoting mental well-being, reinforcing the importance of both physical practice and cognitive understanding in stress reduction.

We observed a significant dropout rate in our tele-yoga intervention trial, with a 63% attrition rate by the 16-week mark. As highlighted in the results section (Table 1), a likely reason for this high dropout rate is that participants who withdrew from the study reported significantly fewer psychological and general health issues compared to those who completed the trial. Given that the participants were predominantly working professionals in a productive age range (mean age 37.38 ± 13.19 years), it is possible that those experiencing less severe anxiety, depression, and insomnia felt considerable improvements after just 4 weeks of yoga practice. As noted in section 3.2, participants who dropped out after four weeks experienced a greater reduction in stress levels than those who continued from week four to week sixteen. Notably, attrition at this early stage was only 33%, with 29 participants dropping out at the 4-week assessment. As these individuals experienced improvements in their mental health, they might have become busier with their work commitments and less inclined to continue the intervention. Another potential reason for increased attrition could be the sharing of the YouTube links of the yoga modules by the authors during the study. Having access to these links may have led participants to feel less compelled to log in for live sessions, as they could practice at their convenience. To improve adherence in future studies, it would be beneficial to provide these resources only at the end of the live tele-yoga intervention. This approach would encourage participants to prioritize live practices and fully engage with the structured elements of the program. Additionally, incorporating reminders or incentives for attending live sessions could further enhance participation. Our documentation indicated that the primary reason for dropout was a lack of time due to busy schedules, as many subjects were unable to continue or were not reachable for follow-up. Importantly, none of the participants reported any adverse effects from the tele-yoga practice, suggesting that the intervention was safe and well-tolerated. These findings highlight the challenge of maintaining participant engagement in long-term interventions, especially among individuals who may experience quick benefits. Future studies could consider strategies to enhance retention, such as offering flexible scheduling, regular follow-ups, or incorporating elements that encourage ongoing participation. Understanding the factors that influence dropout rates is essential for optimizing tele-yoga programs and ensuring their accessibility and effectiveness for those in need.

Yoga reduces stress through several mechanisms, with one key pathway being the down-regulation of the hypothalamic–pituitary–adrenal (HPA) axis, leading to reduced cortisol levels. Studies have shown that both acute sessions and long-term practice (up to 6 months) can significantly lower cortisol levels in individuals with psychiatric disorders (Thirthalli et al., 2013; Bershadsky et al., 2014). Psychological models further elucidate the benefits of yoga through four primary mechanisms: enhanced relaxation, increased mindfulness, improved sense of connectedness, and heightened compassion towards oneself and others (Kishida et al., 2018). Enhanced relaxation fosters emotional regulation and mitigates anxiety, while mindfulness promotes present-moment awareness, reducing ruminative thought patterns. The sense of connectedness cultivated through yoga encourages social support, which can alleviate feelings of isolation. Finally, developing compassion contributes to healthier relationships and greater emotional resilience. These mechanisms underscore the multifaceted nature of yoga as an effective intervention for stress reduction, integrating both physiological and psychological dimensions.

The findings of this study support the need for further research into tele-yoga as a viable tool in mental health services, particularly for disaster management. Tele-yoga offers a cost-effective and scalable intervention that can be easily implemented across various settings. However, the current study has several limitations: (1) Without a control arm, we cannot draw definitive conclusions regarding the efficacy of the module on the specified variables; (2) While the feasibility study was designed to assess both the shorter and longer versions of the tele-yoga modules for stress management, the present study design does not allow for the segregation of the effects of the different yoga modules.

Future studies should consider employing larger sample sizes and more robust methodologies, as well as integrating objective assessment tools to enhance the validity of the findings. Future trials should also focus on differentiating the effects of the longer and shorter versions of the yoga module on efficacy, safety, and attrition. Additionally, investigating the impact of yoga philosophical discussions on participant engagement and overall outcomes could provide valuable insights into how to optimize tele-yoga programs. By exploring these avenues, researchers can better understand the potential of tele-yoga to improve mental health in diverse populations, especially in the context of crisis situations.

## 5 Conclusion

Tele-yoga has demonstrated potential as an effective intervention to mitigate stress, anxiety, and insomnia in individuals who have experienced COVID-19 infection within the past year and report moderate levels of psychological stress. Both the longer (50 min) and shorter (25 min) versions of the tele-yoga module were found to be feasible for the adult population experiencing stress. Future research should further investigate the utility of tele-yoga as a component of mental health services, particularly in the context of disaster management, to enhance the well-being of affected populations.

## Data Availability

The original contributions presented in the study are included in the article/supplementary material, further inquiries can be directed to the corresponding author.

## Footnotes

^1^ https://yoga.ayush.gov.in/ministryofayush/1595399247.pdf

^2^ https://youtu.be/Q1DrHu5fk3g

^3^ https://youtu.be/AnaG04oq4k8

^4^ https://youtu.be/4VPKnXkGR64

^5^See footnotes 2 and 3.
